# Supplementary material for: High Temperature Alters Anthocyanin Concentration and Composition in Grape Berries of Malbec, Merlot, and Pinot Noir in a Cultivar-Dependent Manner
Source: Plants (Basel). 2022 Mar 30;11(7):926. doi: 10.3390/plants11070926 (PMC9003205; doi:10.3390/plants11070926)
Supplement: Supplementary file 1 [file plants-11-00926-s001.zip › plants-1639913-supplementary.pdf]

SUPPLEMENTARY MATERIALS

High temperature alters anthocyanin concentration and composition in grape berries of Malbec, Merlot, and Pinot Noir in a cultivar-dependent manner

Inés de Rosas, Leonor Deis, Yésica Baldo, Juan B. Cavagnaro, Pablo F. Cavagnaro

Supplementary Table S1. Concentration of nine anthocyanin pigments in berries of Malbec, Merlot, and Pinot Noir plants grown under increased (HT) and natural –control-temperature (C) conditions, during the fruit ripening process. Df, delphinidin-3-glucoside; Cn, cyanidin-3-glucoside; Pt, petunidin-3-glucoside; Po, peonidin-3-glucoside; Mv, malvidin-3-glucoside; PoAc, peonidin-3-O-acetylglucoside; MvAc, malvidin-3-O-acetyl-glucoside; PoCu, peonidin-3-O-coumaroyl-glucoside; MvCu, malvidin-3-O-coumaroyl-glucoside. Values are means ± standard errors of four replicates, expressed as µg per g of berry skin ($\mu g \cdot g^{-1}$ FW). Compounds significantly affected by the temperature treatments are denoted in bold letters, and their percentual decrease in concentration (% reduction) indicated. Asterisks indicate significant difference between HT and C treatments at $p \leq 0.05$ (*), $p \leq 0.01$ (**), and $p \leq 0.001$ (***), for a given year, phenological stage, and cultivar (DGC test).

Table S1.

Anthocyanin/ Year	MALBEC								
	Veraison			Half ripeness			Harvest		
	High Temperature	Control	% reduction	High Temperature	Control	% reduction	High Temperature	Control	% reduction
**Df**									
2017	**37.7 ± 5.5**	**83.5 ± 9.7****	50.0	**159.6 ± 16.1**	**236.9 ± 40.8***	33.3	187.2 ± 39.1	187.3 ± 33.8	
2018	**77.1 ± 13.2**	**127.0 ± 8.6***	38.5	136.5 ± 31.0	150.8 ± 11.0		144.6 ± 20.3	162.6 ± 9.6	
**Cn**									
2017	**8.2 ± 1.2**	**20.6 ± 3.1***	50.0	42.6 ± 3.4	42.8 ± 10.8		40.7 ± 5.0	34.9 ± 2.9	
2018	**9.1 ± 0.8**	**18.7 ± 1.7***	50.0	12.2 ± 3.4	11.0 ± 2.1		2.9 ± 0.6	3.3 ± 0.6	
**Pt**									
2017	**72.0 ± 8.4**	**159.1 ± 14.8****	56.3	332.3 ± 27.7	426.9 ± 77.0		354.7 ± 67.8	348.5 ± 59.5	
2018	**127.5 ± 20.0**	**201.2 ± 17.7***	35.0	239.7 ± 51.3	262.3 ± 15.7		265.6 ± 33.9	297.5 ± 14.1	
**Po**									
2017	**32.6 ± 5.4**	**66.6 ± 8.5***	57.1	**218.8 ± 20.5****	**166.5 ± 25.2**	22.7	**288.3 ± 37.7***	**172.1 ± 14.3**	41.4
2018	**50.0 ± 5.6**	**80.9 ± 4.8***	37.5	57.9 ± 12.0	75.8 ± 3.7		**9.3 ± 0.3**	**61.0 ± 26.1***	83.3
**Mv**									
2017	**399.1 ± 41.4**	**864.3 ± 63.8*****	53.5	1916.1 ± 115.7	2181.2 ± 337.9		2306.5 ± 376.4	2108.6 ± 292.0	
2018	**620.6 ± 81.8**	**912.0 ± 112.6***	31.9	1431.4 ± 260.0	1516.5 ± 60.3		1811.3 ± 222.1	2002.9 ± 50.1	
**PoAc**									
2017	**2.4 ± 0.4**	**5.5 ± 0.6****	52.0	**8.4 ± 0.9***	**6.8 ± 1.63**	19.0	**5.3 ± 0.7***	**3.5 ± 0.4**	34.0
2018	2.4 ± 0.2	2.6 ± 0.1		5.1 ± 0.5	4.5 ± 0.7		4.7 ± 0.4	4.3 ± 0.2	
**MvAc**									
2017	**42.8 ± 5.3**	**101.0 ± 7.9****	60.0	106.2 ± 10.1	128.1 ± 30.9		58.4 ± 11.5	52.0 ± 6.2	
2018	8.6 ± 1.3	17.8 ± 5.7		**18.3 ± 4.7***	**7.0 ± 2.7**	50.0	4.8 ± 0.7	5.5 ± 0.3	
**PoCu**									
2017	**23.9 ± 2.2**	**45.8 ± 4.0****	60.0	**63.4 ± 5.8***	**49.0 ± 7.8**	16.7	**92.0 ± 9.4****	**56.4 ± 7.0**	33.3
2018	**21.9 ± 1.1**	**31.1 ± 0.8***	33.3	39.9 ± 4.4	37.2 ± 1.8		52.8 ± 3.9	47.5 ± 3.7	
**MvCu**									
2017	**236.9 ± 24.2**	**516.6 ± 48.2****	53.8	637.4 ± 11.8	702.4 ± 99.7		917.1 ± 113.9	829.3 ± 92.4	
2018	223.1 ± 20.9	310.6 ± 45.7		**860.3 ± 83.4***	**727.6 ± 60.6**	15.1	1019.8 ± 107.8	1015.3 ± 46.3	

Table S1. *continued.*

	MERLOT								
	Veraison			Half ripeness			Harvest		
Anthocyanin/ Year	High Temperature	Control	% reduction	High Temperature	Control	% reduction	High Temperature	Control	% reduction
**Df**									
2017	40.8 ± 8.3	26.5 ± 8.2		**157.8 ± 15.8**	**218.4 ± 23.1***	27.3	233.2 ± 81.2	181.4 ± 30.9	
2018	81.2 ± 4.3	89.3 ± 16.9		**65.0 ± 19.2**	**138.2 ± 34.2***	50.0	133.9 ± 21.3	179.9 ± 19.8	
**Cn**									
2017	20.0 ± 3.3	14.3 ± 4.9		84.2 ± 6.7	94.5 ± 5.8		**150.3 ± 25.1***	**83.6 ± 15.3**	46.7
2018	19.0 ± 2.7	28.4 ± 10.0		**10.7 ± 3.9**	**29.1 ± 8.0***	66.7	**23.3 ± 4.5**	**48.4 ± 5.9***	60.0
**Pt**									
2017	65.6 ± 11.7	43.6 ± 11.4		**271.8 ± 22.1**	**326.0 ± 28.3***	18.2	311.6 ± 90.7	274.2 ± 36.7	
2018	117.5 ± 7.1	95.61 ± 16.84		**93.9 ± 22.1**	**172.3 ± 32.2***	47.1	190.1 ± 27.1	236.3 ± 23.2	
**Po**									
2017	70.7 ± 9.6	54.5 ± 14.6		261.9 ± 22.9	247.1 ± 17.7		**379.5 ± 71.1***	**252.7 ± 23.0**	34.2
2018	61.9 ± 4.0	63.4 ± 15.9		**41.5 ± 11.8**	**99.6 ± 22.0***	60.1	**99.2 ± 16.4**	**159.7 ± 12.7***	37.5
**Mv**									
2017	**385.9 ± 60.0***	**238.4 ± 50.2**	38.5	1400.7 ± 116.6	1385.0 ± 48.9		1425.8 ± 350.4	1413.8 ± 144.2	
2018	**574.6 ± 83.3***	**344.1 ± 38.5**	40.4	**540.5 ± 74.1**	**778.2 ± 60.7***	30.8	1247.8 ± 133.2	1295.7 ± 121.2	
**PoAc**									
2017	6.3 ± 0.8	4.7 ± 1.3		11.9 ± 1.2	12.5 ± 0.7		5.7 ± 0.6	5.5 ± 0.5	
2018	**1.3 ± 0.2***	**0.6 ± 0.1**	53.0	1.3 ± 0.1	1.2 ± 0.1		**2.3 ± 0.3***	**1.5 ± 0.1**	36.9
**MvAc**									
2017	**51.2 ± 7.8***	**30.4 ± 6.7**	40.0	92.6 ± 16.1	100.2 ± 4.6		35.7 ± 4.6	47.8 ± 3.1	
2018	**2.6 ± 0.4***	**0.8± 0.2**	69.0	1.4 ± 0.6	1.37 ± 0.3		8.5 ± 0.9	7.1 ± 1.3	
**PoCu**									
2017	**9.6 ± 1.6***	**6.3 ± 1.9**	15.9	29.9 ± 3.1	24.5 ± 2.4		**58.4 ± 11.9***	**42.4 ± 2.9**	33.3
2018	11.4 ± 1.0	9.5 ± 1.6		11.7 ± 3.1	17.8 ± 2.0		**26.5 ± 3.9**	**38.5 ± 3.9***	25.0
**MvCu**									
2017	**64.5 ± 11.4***	**40.0 ± 10.7**	33.3	164.4 ± 14.2	150.8 ± 1.4		215.1 ± 51.5	232.1 ± 29.2	
2018	**88.0 ± 19.2***	**45.9 ± 3.9**	44.4	106.5 ± 13.9	132.0 ± 6.0		252.2 ± 28.4	242.8 ± 25.8	

Table S1. ***continued.***

Anthocyanin/ Year	PINOT NOIR								
	Veraison			Half ripeness			Harvest		
	High Temperature	Control	% reduction	High Temperature	Control	% reduction	High Temperature	Control	% reduction
**Df**									
2017	**10.1 ± 2.7**	**6.4 ± 2.8***	36.7	15.0 ± 1.3	13.9 ± 5.4		23.6 ± 1.5	22.6 ± 1.2	
2018	**4.8 ± 0.9**	**15.9 ± 5.2***	70.3	**17.4 ± 5.4**	**43.4 ± 1.7***	50.0	**45.4 ± 7.4**	**62.5 ± 4.8***	16.7
**Cn**									
2017	4.0 ± 0.3	2.8 ± 0.9		9.5 ± 0.9	8.5 ± 3.3		19.2 ± 2.4	16.6 ± 3.2	
2018	3.1 ± 1.1	5.5 ± 2.2		11.5 ± 2.6	12.8 ± 0.3		**26.8 ± 7.2**	**40.9 ± 1.4***	25.0
**Pt**									
2017	61.7 ± 14.3	45.4 ± 14.1		78.4 ± 7.3	68.8 ± 20.3		89.3 ± 2.8	93.9 ± 4.0	
2018	15.6 ± 3.4	36.7 ± 12.2		**46.2 ± 12.1**	**85.1 ± 4.2***	44.4	**86.1 ± 10.1**	**108.5 ± 4.6***	18.2
**Po**									
2017	137.1 ± 14.7	156.2 ± 14.5		219.5 ± 32.6	238.0 ± 49.6		328.3 ± 5.3	349.8 ± 42.7	
2018	66.7 ± 30.3	61.52 ± 16.10		165.7 ± 41.4	173.1 ± 10.2		**318.1 ± 74.8**	**417.9 ± 22.9***	23.1
**Mv**									
2017	1302.1 ± 196.9	1247.6 ± 123.2		1454.6 ± 141.1	1400.5 ± 167.4		**1411.9 ± 38.1**	**1662.6 ± 60.1***	15.1
2018	292.4 ± 79.3	392.8 ± 102.7		641.6 ± 156.8	811.7 ± 55.5		**953.8 ± 74.7**	**1136.4 ± 43.0***	16.7
**PoAc**									
2017	ND	ND		ND	ND		ND	ND	
2018	ND	ND		ND	ND		ND	ND	
**MvAc**									
2017	ND	ND		ND	ND		ND	ND	
2018	ND	ND		ND	ND		ND	ND	
**PoCu**									
2017	ND	ND		ND	ND		ND	ND	
2018	ND	ND		ND	ND		ND	ND	
**MvCu**									
2017	ND	ND		ND	ND		ND	ND	
2018	ND	ND		ND	ND		ND	ND	

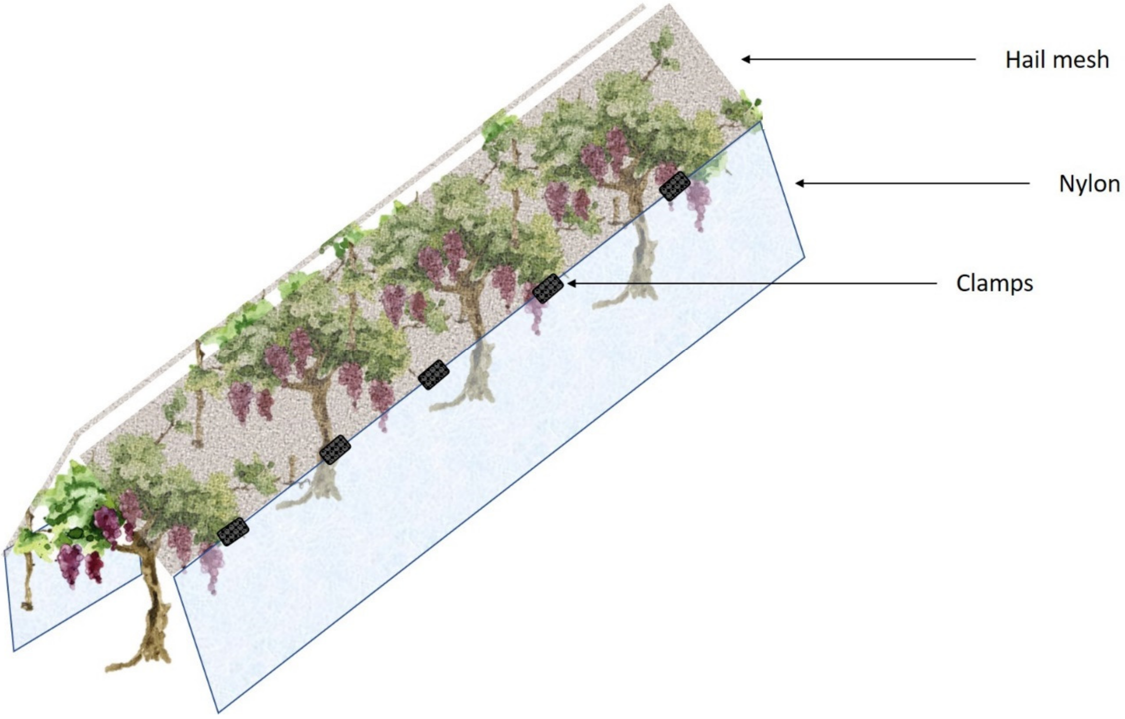

Supplementary Figure S1. Nylon structure used to increase temperature at berry site in the high temperature (HT) treatment.

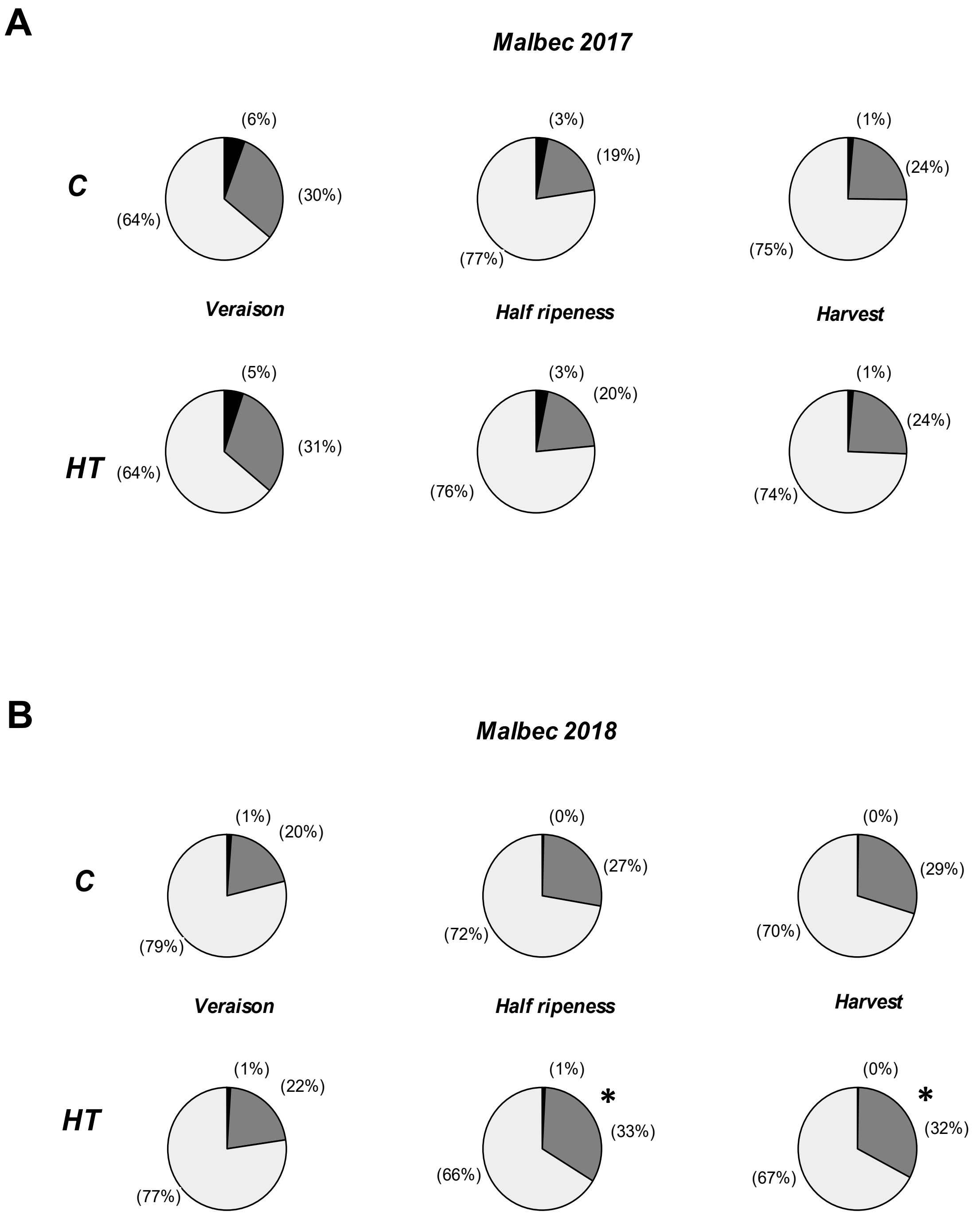

Supplementary Figure S2. Relative content (%) of acetylated (black), coumarylated (gray), and non-acylated anthocyanins (light gray) in berries of Malbec grown under high temperature (HT) and control (C) conditions, in 2017 (A) and 2018 (B). Data from four replicates are shown. Asterisks indicate statistical differences between non-acylated and acylated anthocyanins (i.e., coumarylated and acetylated combined) at $p \leq 0.05$ between HT and C treatments at a given phenological stage (LSD test).

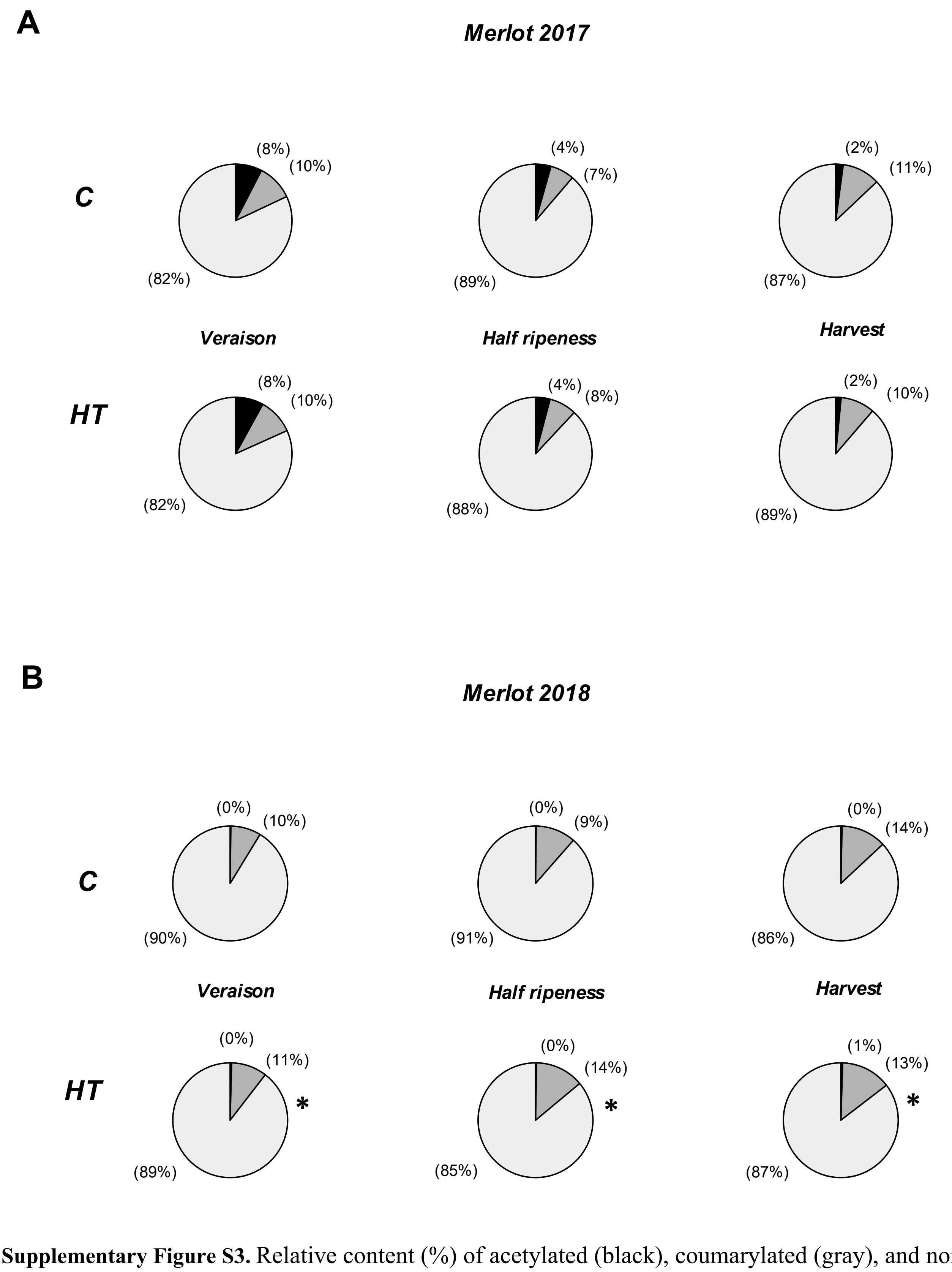

Supplementary Figure S3. Relative content (%) of acetylated (black), coumarylated (gray), and non-acylated anthocyanins (light gray) in berries of Merlot grown under high temperature (HT) and control (C) conditions, in 2017 (A) and 2018 (B). Data from four replicates are shown. Asterisks indicate statistical differences between non-acylated and acylated anthocyanins (i.e., coumarylated and acetylated combined) at $p \leq 0.05$ between HT and C treatments at a given phenological stage (LSD test).

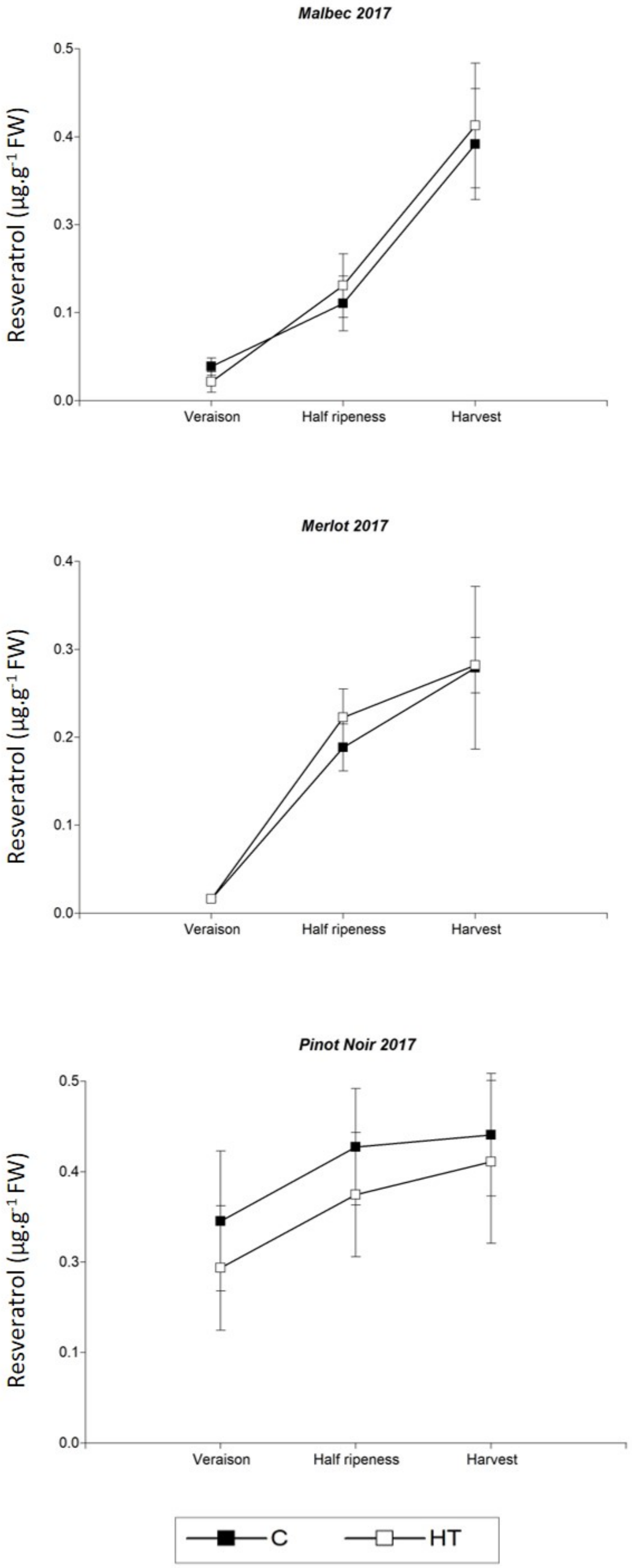

Supplementary Figure S4. *Trans*-resveratrol concentration in Malbec (A), Merlot (B) and Pinot Noir (C) berries grown under high temperature (HT) and control (C) conditions in 2017. Error bars represent standard errors from four replicates. Asterisks indicate statistical significance ($p \leq 0.05$) between treatments according to mean comparison, LSD test. Resveratrol content in 2018 was determined in

berries of the three grape cultivars, but no significant differences were found between the HT and C treatments (data not shown), similarly to 2017.
